# Post-Fire Axial Compressive Behavior of Circular GFRP Tube-Confined Concrete Short Columns

**DOI:** 10.3390/ma19030634

**Published:** 2026-02-06

**Authors:** Yiwei Tang, Liu Yang, Ni Zhang, Yali Feng, Jixiang Li

**Affiliations:** 1 Yangtze University College of Arts and Sciences, Jingzhou 434020, China; 201872475@yangtzeu.edu.cn (L.Y.); zhangni0212@163.com (N.Z.); fengyali2016@hust.edu.cn (Y.F.); 2 School of Civil and Hydraulic Engineering, Huazhong University of Science and Technology, Wuhan 430074, China; 3 School of Civil Engineering and Architecture, Wuhan Polytechnic University, Wuhan 430023, China

**Keywords:** GFRP tube, CFGFT short columns, axial compressive behavior, elevated temperature, residual axial capacity, capacity reduction factor

## Abstract

**Highlights:**

**What are the main findings?**

- Peak temperature governs residual axial capacity and stiffness of CFGFT columns.
- Wall thickness affects confinement efficiency and failure mode after fire exposure.
- Constant temperature duration (60–120 min) shows no systematic strength effect.

**What are the implications of the main findings?**

- Post-fire performance should be assessed based on peak temperature rather than duration.
- Thick GFRP tubes provide only relative confinement advantages at high temperatures.
- A reduction-factor model enables rapid post-fire capacity evaluation of CFGFT members.

**Abstract:**

This study experimentally investigates the residual axial compression behavior of circular glass fiber-reinforced polymer (GFRP) tube-confined concrete short columns (CFGFT) after exposure to elevated temperatures. A total of 27 specimens were fabricated and tested under axial compression, with key parameters including GFRP tube wall thickness (5, 8, and 10 mm), exposure temperature (100, 150, 200, and 300 °C), and constant temperature duration (60 and 120 min). The results show that the load–displacement responses of CFGFT short columns after elevated temperature exposure exhibit distinct two-stage characteristics, culminating in brittle failure at the ultimate axial capacity. Wall thickness significantly influences the failure modes of the specimens, while elevated temperatures increase the occurrence of unfavorable failure modes. Temperature is identified as the primary factor governing the degradation of residual axial capacity and initial stiffness, with performance deterioration becoming more pronounced at temperatures exceeding 200 °C. In contrast, the effect of constant temperature duration within the range of 60–120 min is relatively limited. Based on the experimental results, a simplified binary quadratic regression model incorporating the coupled effects of temperature and wall thickness is proposed to predict the post-fire axial capacity reduction factor (Kr), with a coefficient of determination (R^2^) of 0.901. These findings provide experimental evidence and a practical predictive approach for the fire-resistant design and post-fire safety assessment of CFGFT members.

## 1. Introduction

Fiber-Reinforced Polymer (FRP) has been widely used in fields such as bridges, buildings, marine, and underground engineering in recent years due to its high strength, light weight, excellent corrosion resistance, and ease of construction [1,2]. Among these applications, the Concrete-Filled GFRP Tube (CFGFT), formed by filling pultruded round GFRP tubes with concrete, effectively utilizes the lateral confinement of the GFRP tube on the core concrete. This confinement results in a triaxial compressive stress state for the concrete, significantly enhancing the load-bearing capacity, deformation capacity, and seismic performance of the component [3,4,5]. As a result, CFGFTs have gradually become an important type of compression member in civil engineering [6,7].

However, throughout the structural lifecycle, CFGFT components inevitably face threats from high-temperature environments such as fires. As one of the most severe extreme hazards to building structures, fire induces high-temperature effects that lead to the dual degradation of both material properties and structural performance, severely compromising structural safety. On one hand, concrete, as the primary load-bearing material, exhibits highly temperature-sensitive mechanical properties. Studies show that its compressive strength significantly decreases when temperatures exceed 300 °C [8,9,10]. On the other hand, the resin matrix within GFRP materials softens or even undergoes thermal decomposition under elevated temperatures, weakening the fiber–matrix interface properties. This leads to a rapid decline in the confinement capacity of GFRP tubes on the core concrete. This thermal sensitivity represents an inherent vulnerability that distinguishes CFGFT structures from traditional reinforced concrete members [11]. Therefore, systematically investigating the degradation patterns of CFGFT members’ mechanical properties after elevated temperature exposure holds significant theoretical and engineering value for their fire-resistant design, post-fire safety assessment, and repair/reinforcement.

The effect of temperature on the properties of GFRP materials has been extensively studied at the material scale, with consensus that GFRP is highly sensitive to elevated temperatures and that a clear threshold exists for the degradation of its mechanical properties. Aydin [7] showed that the tensile and compressive strengths of pultruded GFRP profiles decreased by approximately 28% and 67%, respectively, at 100 °C, and their compressive load-bearing capacity was almost completely lost at 200 °C. Khaneghahi et al. [12] further pointed out that the compressive strength of GFRP decreases by more than 50% when the temperature reaches the softening point of the resin (around 90 °C), and that the cross-sectional geometry parameter (the ratio of cross-sectional area to perimeter) is a critical factor influencing its mechanical properties at elevated temperatures. Additionally, the study by Zhang C [13] highlighted the significant effect of cooling methods on the residual properties of GFRP: while air cooling showed some recovery of strength, water cooling resulted in the most severe degradation, with almost complete strength loss after 300 °C. These studies have systematically established the sensitivity of GFRP and its performance degradation characteristics in high-temperature environments at the material scale.

At the component scale, studies on GFRP-reinforced concrete components have revealed the complexity of their post-fire behavior and the limitations of traditional reinforcement methods. Li et al. [14] showed that the concrete protective layer provides a thermal buffer to the internal GFRP reinforcement, allowing it to retain a certain degree of residual properties below 400 °C. However, Khalaf et al. [15] and Martin-Concepcion et al. [16] further pointed out that the mechanical properties of GFRP bars are significantly degraded when the temperature exceeds approximately 200 °C, with their contribution to the axial load capacity of concrete columns as longitudinal reinforcement after a fire being extremely limited. Related studies also show that the thickness of the concrete protective layer and the residual strength of GFRP reinforcement are key factors influencing the fire resistance performance of the members. Moreover, the use of external thermal insulation measures can more effectively enhance the post-fire residual load-carrying capacity of the members, reaching approximately 60% of the load-carrying capacity at room temperature. These findings highlight the inherent limitations of traditional GFRP-reinforced concrete members in terms of fire resistance design.

In recent years, some scholars have begun to focus on the fire performance of circular GFRP tubular concrete columns (CFGFT) and have made preliminary explorations into the effects of geometric parameters and loading conditions. Barber et al. [17] found that increasing the length-to-slenderness ratio of the components can significantly enhance the fire resistance of CFGFT columns, extending the fire resistance time by 235% compared to ordinary concrete columns. Guo et al. [18] and Mollakhalili et al. [19] showed that increasing the thickness of GFRP tubes can improve the ultimate load-bearing capacity of tubular concrete columns, but heating significantly reduces this enhancement. Radhouane Masmoudi et al. [20] pointed out that the strain distribution and high-temperature cracking resistance between FRP tubes and concrete are significantly affected by the ratio of the diameter of the FRP tube to the thickness of the concrete protective layer (D/c). Tabatabaeian et al. [21] investigated the axial ultimate bearing capacity and strain characteristics of GFRP pipes and GFRP pipe–concrete columns after high-temperature exposure. The results indicated that, with increasing temperature and exposure time, both axial bearing capacity and ultimate strain decreased, but the effect of exposure duration was relatively insignificant. Additionally, Morgado et al. [22,23] developed a finite element model to predict the fire resistance of GFRP columns based on experimental tests.

In summary, although the mechanical properties of GFRP composites and CFGFT members under elevated temperatures have been investigated at both the material and component levels (e.g., [18,19,20,21]), most existing studies focus on the effects of one or two parameters, with fewer systematic correlations between the material degradation behavior of GFRP tubes and the overall axial stress performance of the members within a unified experimental framework. Consequently, evidence remains limited for simultaneously quantifying the coupled effects of three parameters—tube wall thickness, peak temperature, and exposure duration—on the post-heating axial behavior of circular CFGFT short columns under a consistent heating–holding–cooling protocol. Moreover, the quantitative degradation relationships and engineering-oriented predictive tools for key post-fire indicators (e.g., residual axial capacity, stiffness, and ductility) are not yet sufficiently established, which restricts fire-resistant design and post-fire safety assessment of CFGFT members.

Based on the above background, this study experimentally investigates the post-heating axial compressive behavior of circular GFRP tube-confined concrete short columns (CFGFT). A total of 27 specimens were tested, considering three key variables: tube wall thickness (5, 8, and 10 mm), peak temperature (100, 150, 200, and 300 °C), and constant-temperature duration (60 and 120 min). The responses are evaluated in terms of failure modes, ultimate load capacity, initial stiffness, and displacement ductility, together with representative strain-based confinement responses. On this basis, the degradation of GFRP confinement after thermal exposure is discussed, and a practical reduction-factor-based model is proposed and validated to predict the post-fire axial capacity of circular CFGFT short columns. The results provide a benchmark dataset and an assessment-oriented tool for post-fire evaluation of circular CFGFT short columns within the investigated parameter range.

## 2. Materials and Methods

### 2.1. Specimen Design and Preparation

A total of 27 pultruded circular GFRP pipe concrete short column (CFGFT) specimens were designed and fabricated for this study. Of these, 24 specimens were subjected to different high-temperature treatments for varying durations, while the remaining 3 specimens were left untreated as control groups at ambient conditions for performance benchmarking. The three ambient specimens served as thickness-matched baselines, with one reference specimen for each tube wall thickness (5, 8, and 10 mm). Post-fire performance was primarily evaluated using normalized indices (e.g., λ = Nu/N_0_); however, the ambient baseline was defined by a single specimen per thickness, and additional ambient repeats are recommended in future work to improve statistical robustness.

The specimens were designed with uniform dimensions: an outer diameter of 200 mm (D_0_) and a net height of 450 mm (H), resulting in a height-to-diameter ratio (H/D_0_) of 2.25. This ratio is typical for short columns and ensures that damage is controlled primarily by material strength rather than instability.

Three main variables were investigated:

GFRP Pipe Wall Thickness (H_t_): 5 mm, 8 mm, and 10 mm

Temperature (T): 100 °C, 150 °C, 200 °C, and 300 °C (with an additional control group at 20 °C)

Constant Temperature Duration (D): 60 min and 120 min

For clarity, a uniform naming convention is adopted: C-HtX-TY-DZ, where C represents the circular cross-section; X in HtX denotes the wall thickness (5, 8, 10); Y in TY represents the target temperature (100, 200, 300, with 0 indicating ambient temperature); and Z in DZ corresponds to the exposure duration (60, 120, with 0 indicating ambient temperature of approximately 20 °C). For example, specimen C-Ht8-T200-D60 refers to a specimen with a wall thickness of 8 mm, subjected to 200 °C for 60 min. The detailed design parameters for all specimens are summarized in Table 1.

**Figure 1:**
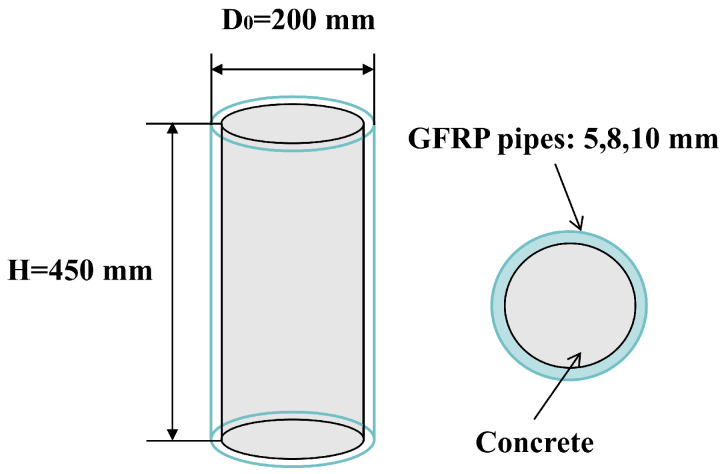
Schematic diagram of the circular CFGFT short column specimen.

In order to prepare the CFGFT short column specimens, the GFRP pipe was first cut to the designed net height, and the opening at the end of the pipe was sanded to ensure a flat surface for reliable loading contact. The bottom of the pipe was then sealed with high-strength adhesive tape to form a temporary bottom mold, preventing slurry leakage during the pouring process. The concrete was mixed according to the established proportions, poured into the pipe in layers, and vibrated on a vibrating table until no visible bubbles or segregation appeared on the surface, ensuring proper compaction. The tops of the specimens were leveled, and they were transferred to a standard curing room (20 ± 2 °C, relative humidity > 95%) for 28 days. During this time, 150 mm cubic standard specimens were also cast and cured under the same conditions to determine the cubic compressive strength of the concrete. Figure 1 shows the schematic diagram of the circular CFGFT short column specimen.

### 2.2. Material Properties

#### 2.2.1. Concrete

The design strength grade of the concrete used in this test is C30, and the mix ratio is shown in Table 2. To determine the compressive strength, standard cubes of 150 mm × 150 mm × 150 mm were cast under the same conditions as the CFGFT short columns and cured for 28 days under standard conditions. An axial compression test was conducted according to the standard test method, and the cubic compressive strength (f_cu_) was measured. The results are summarized in Table 2. This strength was used to determine the strength class of the concrete.

#### 2.2.2. GFRP Pipe

The GFRP pipe used in this study is a pultruded composite material, consisting of a synthetic resin matrix and a glass fiber reinforcement phase (50% volume fraction), with fibers primarily oriented along the axial direction of the pipe. The main mechanical properties of the material are summarized in Table 3.

### 2.3. Test Program and Setup

#### 2.3.1. High Temperature Test

**Figure 2:**
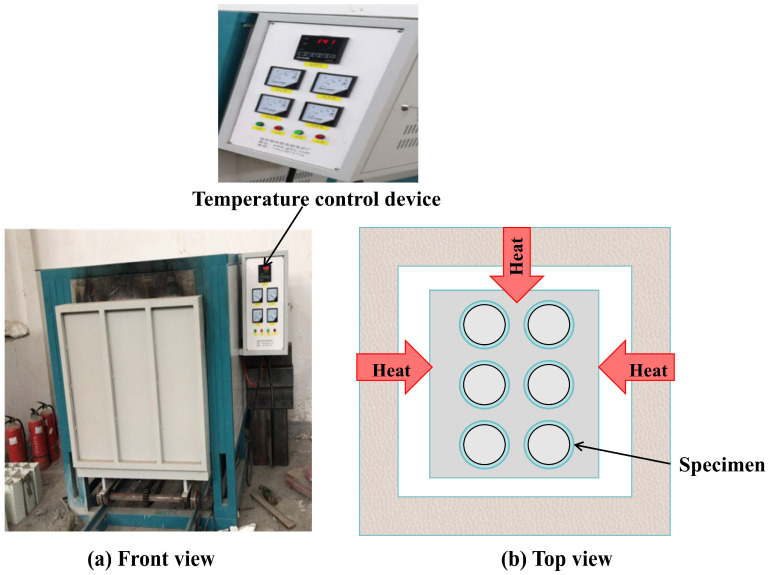
High-temperature furnace and specimen arrangement: (**a**) front view of the furnace; (**b**) top view schematic illustrating multi-directional thermal exposure around the specimens.

To simulate the high-temperature environment experienced by CFGFT columns during a fire, all 24 test groups of specimens were subjected to treatment in a programmed, temperature-controlled high-temperature furnace, as shown in Figure 2. The furnace temperature followed the ISO 834 standard fire heating curve [24], gradually increasing to the preset target temperatures (100, 150, 200, and 300 °C), after which the specimens entered the constant temperature phase for either 60 min or 120 min. The upper bound of 300 °C was selected to represent an engineering-relevant post-fire exposure window for structural members and was also guided by prior heating programs on GFRP tube-confined concrete stub columns adopting ISO 834-based procedures (e.g., [25]). This temperature range also covers the region where the polymer matrix of GFRP approaches or exceeds its glass transition temperature (Tg), depending on the resin system, beyond which resin softening may rapidly reduce composite stiffness/strength and confinement effectiveness. As illustrated in Figure 2, the specimens were placed upright in the furnace chamber and exposed to the thermal environment from multiple directions; to reduce boundary effects, they were positioned with clearance from the furnace walls, although some thermal non-uniformity may still exist during furnace heating. Temperature gradients through the tube thickness or along the specimen height were not directly measured or quantitatively estimated in this study.

During the experiment, temperature was monitored by the furnace’s built-in control system, which displayed real-time readings. No thermocouples were used for direct specimen measurements. Instead, the furnace’s PID control system maintained the temperature, with an actual deviation of ±5 °C from the displayed value. It should be noted that this study focuses on post-heating residual assessment; therefore, the selected temperatures are intended to represent the thermal exposure of structural members rather than the peak hot-gas temperature in a fully developed compartment fire.

At the end of the constant temperature phase, the heat source was turned off, and the specimens were allowed to cool naturally to ambient temperature (approximately 20 °C) inside the furnace before being removed. The three ambient control specimens (T0-D0) were not subjected to this high-temperature treatment. The high-temperature process consisted of three stages: heating, constant temperature, and natural cooling.

#### 2.3.2. Axial Compression Test and Data Acquisition

After cooling, all specimens were tested in axial compression using a microcomputer-controlled electro-hydraulic servo universal testing machine. Prior to the formal loading, the specimens were preloaded (approximately 10% of the estimated ultimate load capacity) to ensure proper alignment and tight contact.

Monotonic static loading, controlled by displacement, was applied at a constant rate of 0.5 mm/min. The test continued until there was a significant reduction in the load-bearing capacity of the specimen or the tearing of the GFRP pipe. Axial load and overall displacement were continuously monitored and recorded in real time by the transducer attached to the testing machine, and the load-displacement curves were plotted accordingly.

**Figure 3:**
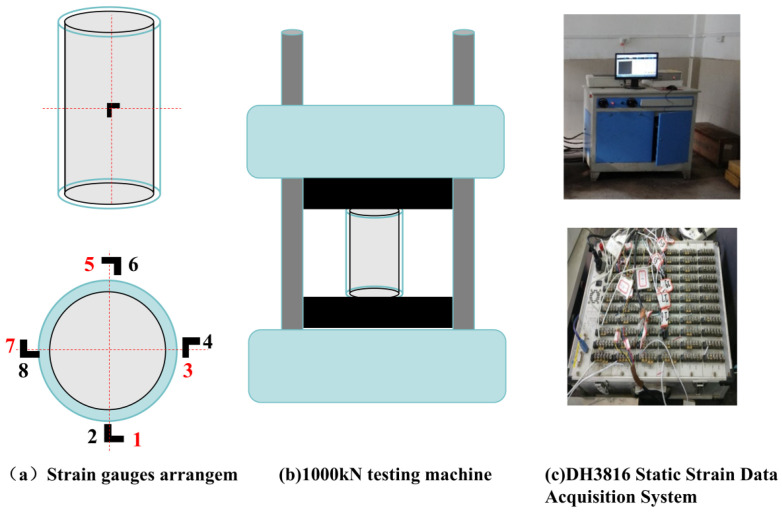
Schematic of the axial compression test setup and strain gauge arrangement.

To thoroughly analyze the strain development, nine representative specimens (CHt5-T0-D0, CHt8-T0-D0, CHt10-T0-D0, CHt5-T100-D120, CHt8-T100-D120, CHt8-T150-D120, CHt8-T200-D120, CHt8-T300-D120, CHt10-T300-D120) were selected for detailed strain measurements. This representative subset was selected to cover the key comparisons in this study under practical instrumentation constraints; similar representative-specimen strategies have been adopted in related elevated-temperature studies on GFRP tube-confined concrete members (e.g., [21,25]). As shown in Figure 3, four measurement points were evenly distributed along the circumferential direction on the outer wall of the central section of each specimen, spaced at 90° intervals. At each measurement point, one longitudinal and one circumferential strain gauge were attached to monitor the axial compression and circumferential expansion deformations, respectively. This resulted in a total of eight strain gauges per specimen. The strain data were automatically collected by the DH3816 static strain acquisition system, synchronized with the load and displacement signals. The load-strain curve was then generated based on these measurements.

## 3. Results

### 3.1. Apparent Observations and Failure Modes

#### 3.1.1. Apparent Characteristics After High-Temperature Treatment

After high-temperature treatment, the apparent color and surface state of the GFRP pipes in CFGFT specimens exhibited clear and distinguishable changes depending on the treatment temperature. The effect of high-temperature duration and wall thickness was found to be relatively minor.

At low to moderate temperatures (100 °C and 150 °C), the appearance of the specimens was almost identical to that at room temperature. Even with an extended exposure time of 120 min, no significant thermal oxidation, blistering, or cracking was observed on the surface of specimens with different wall thicknesses (5 mm, 8 mm, and 10 mm). This suggests that the temperature range has not yet caused noticeable thermal damage to the surface layer of the GFRP tubes.

When the temperature reached 200 °C, the surface of the specimens exhibited clear signs of thermal oxidation. The GFRP tubes turned light yellow, with the discoloration mainly occurring in the mid and upper sections of the tubes. The color transition followed a gradient from bottom to top along the height of the tubes. Extending the constant temperature duration from 60 to 120 min only resulted in a slight deepening of the color, and the overall change was limited.

At 300 °C, the thermal damage to the GFRP tubes became significantly more pronounced, with a clear dependence on wall thickness. The 5 mm thin-walled specimens exhibited a light yellow hue, the 8 mm specimens showed more evident thermal oxidation, though the color remained lighter after cooling, while the 10 mm thick-walled specimens underwent a transition from light yellow to dark yellow, with some regions showing slight black charring. Extending the constant temperature exposure to 120 min further intensified the color change and the spread of localized charring patches.

**Figure 4:**
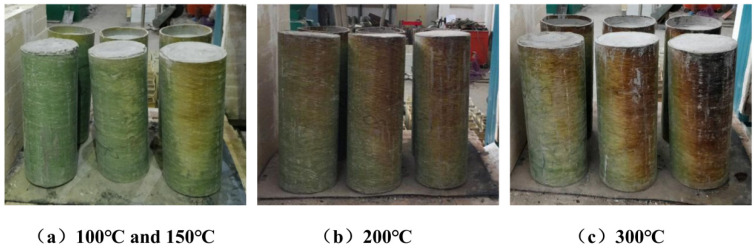
Schematic diagram of the specimen after high temperature.

In general, the apparent thermal damage of GFRP pipes is primarily governed by the treatment temperature, with a significant increase in damage observed above 200 °C. The duration of high temperature exposure mainly influences the extent of color change and its uniformity. Wall thickness also plays a role in the degree of apparent damage under elevated temperature conditions. All specimens maintained their structural integrity after high-temperature treatment, without any macroscopic deformation. These observations are based on visual inspection and photographs; no microscopy, mass-loss, or residual material tests were conducted. Their typical appearance is shown in Figure 4.

#### 3.1.2. Damage Modes

**Figure 5:**
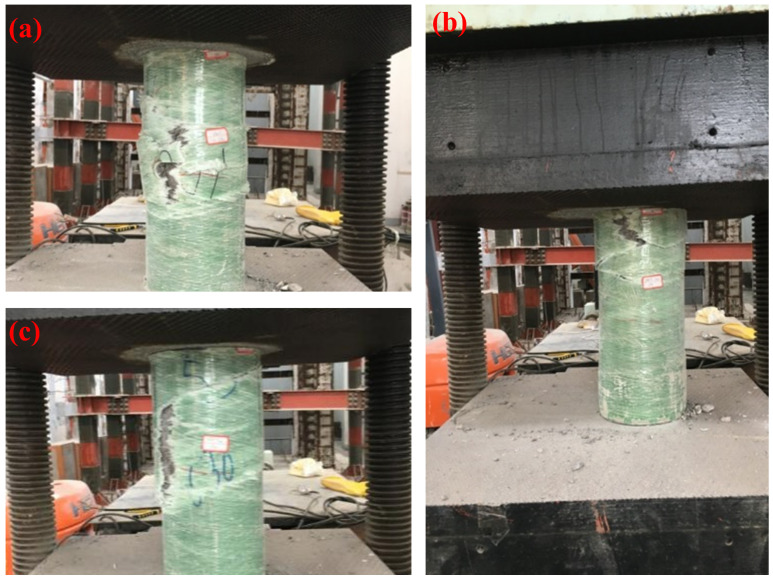
Typical failure modes of GFRP-confined concrete specimens after axial compression: (**a**) circumferential fracture in the middle region; (**b**) terminal compression failure; (**c**) longitudinal splitting failure.

Under axial compression, all specimens ultimately exhibited brittle failure of the GFRP pipe, accompanied by core concrete crushing. Based on the variations in crack initiation and propagation paths, the damage modes of the specimens can be classified into three typical types, as illustrated in Figure 5.

Mode 1: Circumferential fracturein the middle

This is the most common failure mode, where the crack typically initiates in the cen-tral part of the specimen, and the GFRP pipe fails circumferentially. This is often accom-panied by a distinct sound of fiber rupture, followed by crushing of the core concrete and outward bulging. This pattern was predominantly observed in specimens with wall thicknesses of 5 mm and 8 mm. Specimens subjected to high temperatures, particularly at 300 °C, exhibited more abrupt and brittle circumferential fractures.

Mode 2: Terminal compression

Approximately 33% of the specimens displayed localized damage at the loaded end, manifesting as collapse of the GFRP pipe at the end, accompanied by diagonal cracks ex-tending axially toward the center. This failure mode was more prevalent in specimens with 10 mm thick walls. After high-temperature exposure, the end regions were more prone to localized damage, with a further increase in the concentration of damage.

Mode 3: Longitudinal Splitting

A smaller proportion of specimens (around 24%) exhibited longitudinal splitting damage, typically initiating at the upper or lower center of the specimen and rapidly propagating along the axial direction, splitting the tube into several pieces. The higher in-cidence of this mode in high-temperature specimens indicates that elevated temperatures reduce the overall restraining capacity of the GFRP tubes, making them more susceptible to longitudinal splitting.

The damage modes were classified based on macroscopic observations during loading and post-test photographic records (e.g., crack initiation location, propagation direction, and tube rupture pattern); no predefined quantitative thresholds (e.g., crack width, axial shortening, or strain limits) were used as strict criteria in the present study.

In general, wall thickness is the primary factor influencing the damage mode: thin-walled specimens are more likely to exhibit overall central circumferential fractures, while thick-walled specimens are more prone to localized end compression failure. Temperature primarily affects the brittleness of the failure process, with all types of damage modes displaying more abrupt failure characteristics as the treatment temperature increases. These damage patterns provide an intuitive basis for the subsequent analysis of the degradation of axial compressive properties in CFGFT short columns under high-temperature conditions.

### 3.2. Load-Displacement Response and Key Mechanical Parameters

#### 3.2.1. Typical Characteristics of Load-Displacement Curves

**Figure 6:**
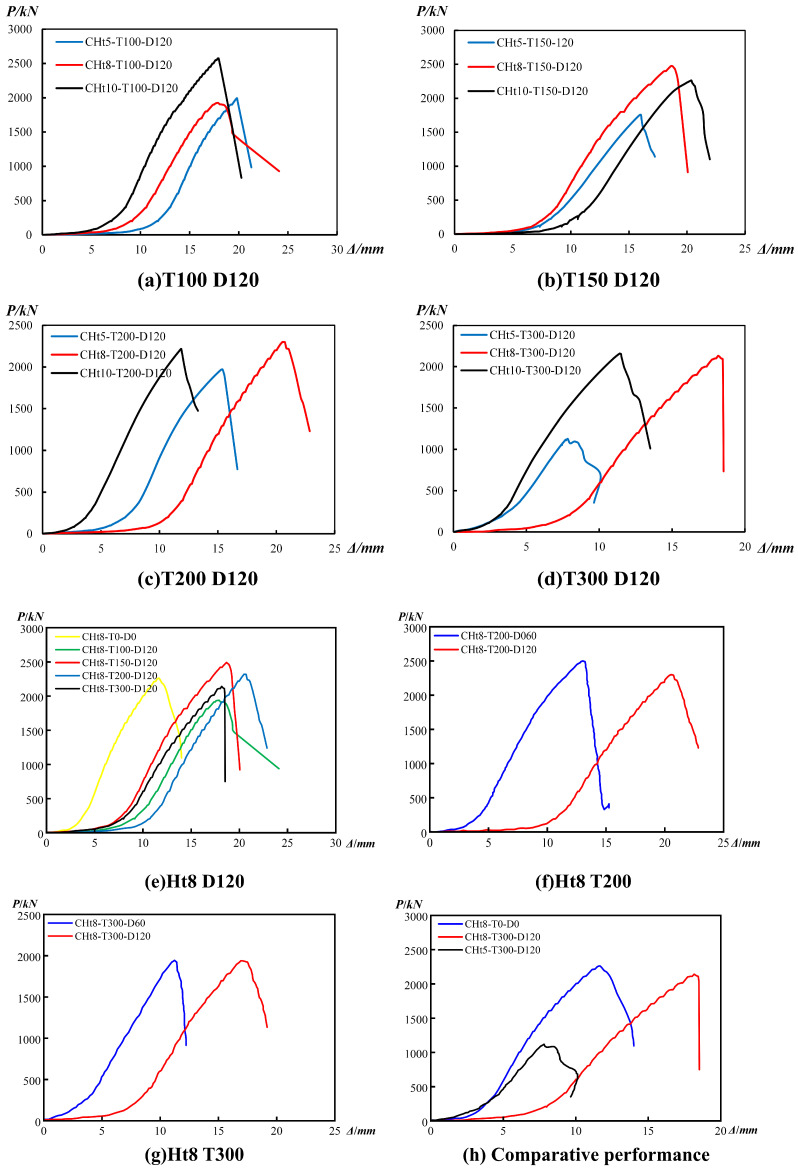
Typical axial load–displacement curves of circular CFGFT stub columns after elevated temperatures: (**a**–**d**) effect of GFRP tube wall thickness at different temperatures (D = 120 min); (**e**) effect of temperature at a constant wall thickness of 8 mm (D = 120 min); (**f**,**g**) effect of high-temperature duration at 200 °C and 300 °C (wall thickness = 8 mm); (**h**) comparison of representative ambient and elevated-temperature conditions.

The axial load–displacement responses of all CFGFT short column specimens exhibit a consistent two-stage (quasi-bilinear) trend, comprising an initial approximately linear elastic stage followed by a progressively nonlinear stage with stiffness degradation and terminating in brittle failure (typical responses are shown in Figure 6). Across the investigated parameter range, degradation of the ultimate load-carrying capacity and deformation characteristics is primarily governed by the peak exposure temperature, while the effects of wall thickness and high-temperature duration play secondary roles.

At relatively low temperatures (100 °C and 150 °C; Figure 6a,b), increasing the GFRP tube wall thickness leads to higher peak loads and steeper initial slopes, resulting in fuller load–displacement responses. This trend reflects the effective confinement provided by thicker GFRP tubes under moderate thermal exposure. When the temperature rises to 200 °C and 300 °C (Figure 6c,d), however, the differences among specimens with different wall thicknesses become much less pronounced. At 300 °C, for example, the ultimate load capacities of the 10 mm and 8 mm specimens are nearly identical, indicating that the contribution of wall thickness to axial compressive performance is substantially reduced at extreme temperatures.

Conditional on wall thickness, temperature exerts a dominant influence on the overall response. As shown in Figure 6e, specimens with an 8 mm wall thickness and a constant temperature duration of 120 min display a progressive contraction of the load–displacement curves as temperature increases. The peak load decreases from 2254 kN at ambient conditions to 2131 kN at 300 °C, accompanied by a reduction in elastic stiffness, peak displacement, and post-peak deformation capacity. Minor fluctuations in ultimate load capacity observed between 150 °C and 200 °C for individual specimens can be attributed to material variability and non-uniform thermal damage, without altering the overall degradation trend.

By contrast, extending the high-temperature duration from 60 min to 120 min produces only limited changes in the load–displacement response (Figure 6f,g). Under the same temperature level, differences in curve shape, peak load, and initial stiffness remain small, suggesting that most thermal degradation occurs during the early stage of exposure rather than during prolonged heating.

Figure 6h further compares representative extreme conditions, including the ambient reference state, high-temperature exposure with constant wall thickness, and combined thin-wall–high-temperature exposure. The results show that exposure to 300 °C is sufficient to induce pronounced degradation in axial compressive performance, with the thin-wall–high-temperature combination representing the most unfavorable condition in terms of load capacity and stiffness.

Within the present test range, these observations indicate that peak temperature defines the primary degradation boundary for CFGFT short columns, while wall thickness mainly affects performance at lower temperatures and exposure duration plays a limited role once thermal damage is established.

The ultimate bearing capacity, initial stiffness, and deformation characteristics of each specimen are systematically summarized in Table 4, providing the necessary data foundation for subsequent quantitative analysis and modeling.

The ductility coefficient (μ) is introduced to evaluate the deformation capacity of CFGFT short columns prior to reaching their ultimate load capacity. It is defined as the ratio of the ultimate axial displacement (Δ_u_) to the yield displacement (Δ_y_) [26], expressed as follows:

μ = Δ_u_/Δ_y_ (1)

Both Δ_u_ and Δ_y_ are obtained from the axial load–displacement curves. The yield displacement Δ_y_ is determined using the secant stiffness method, which is appropriate for composite members exhibiting nonlinear compressive behavior. Δ_u_ represents the ultimate displacement, corresponding to the displacement at the peak axial load (i.e., the ultimate load capacity). Based on these parameters, the post-fire ductility of circular CFGFT short columns is quantitatively evaluated.

**Figure 7:**
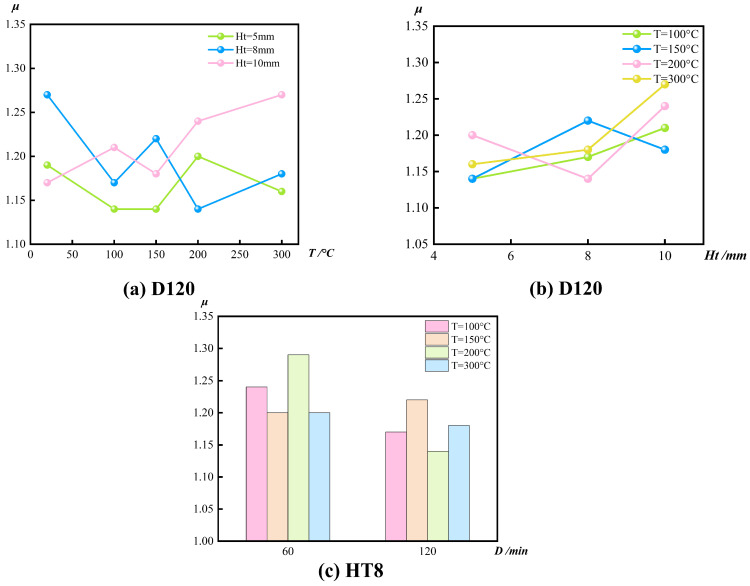
Variation of ductility coefficient of CFGFT stub columns after elevated temperatures.

According to Table 4, the displacement ductility coefficient μ of all specimens remains within a relatively narrow range (1.11–1.29), and its variation with key parameters is summarized in Figure 7. In contrast to the pronounced degradation in ultimate load capacity, μ does not exhibit a clear monotonic decreasing trend with increasing exposure temperature.

For the Ht = 8 mm series at D = 120 min (Figure 7a), μ varies only moderately with temperature, changing from 1.27 under ambient conditions to 1.17, 1.22, 1.14, and 1.18 after exposure to 100, 150, 200, and 300 °C, respectively. This indicates that, within the present test scope, the ductility index is less temperature-sensitive than strength-related indicators.

When interpreted together with ultimate capacity, however, a different implication emerges: moderate μ values can coexist with substantial strength loss. For instance, for the Ht = 5 mm specimens at 300 °C and 120 min, μ remains 1.16, whereas the ultimate load decreases from 2128 kN to 1127 kN. This suggests that an apparently “stable” μ does not necessarily reflect preservation of load-carrying resistance after thermal exposure.

The influence of wall thickness on μ depends on temperature (Figure 7b). At 100 °C (D = 120 min), μ changes only slightly with wall thickness. At higher temperatures, the thickness effect becomes more noticeable; for example, at 200 °C, μ is 1.20, 1.14, and 1.24, and at 300 °C it is 1.16, 1.18, and 1.27 for H_t_ = 5, 8, and 10 mm, respectively. Overall, under severe thermal exposure, thicker tubes tend to show slightly higher μ, reflecting the coupled evolution of confinement effectiveness and deformation development.

As shown in Figure 7c (H_t_ = 8 mm), increasing the constant temperature duration from 60 min to 120 min does not lead to a consistent change in μ. The differences are small at 150 °C and 300 °C (both 0.02), but can be larger at certain temperatures (e.g., 100 °C: 1.24 → 1.17, and 200 °C: 1.29 → 1.14). These case-dependent variations indicate that, within 60–120 min, exposure duration may cause fluctuations in μ, yet it does not form a stable, repeatable trend comparable to the temperature-driven degradation in strength.

Within the investigated parameter range, elevated temperature does not cause systematic deterioration of μ. From a post-fire evaluation perspective, μ should be interpreted together with residual load-bearing capacity (λ), since ductility indices alone may mask the extent of strength degradation induced by thermal exposure.

#### 3.2.2. Initial Stiffness

**Figure 8:**
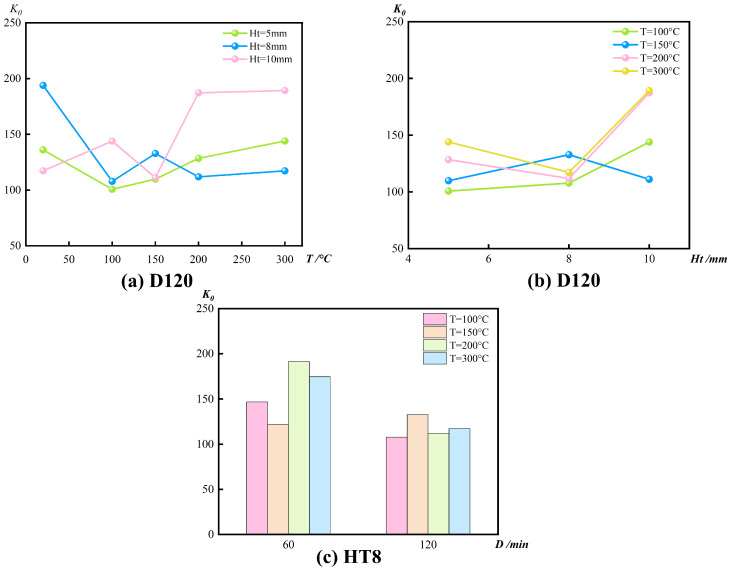
Variation of initial stiffness with key parameters.

Figure 8 illustrates the variation of the initial stiffness K_0_ of circular CFGFT stub columns with exposure temperature, GFRP tube wall thickness, and constant temperature duration. Across all test conditions, temperature is the primary factor governing stiffness degradation, while wall thickness and exposure duration act as secondary modifiers of this process.

For specimens with a wall thickness of 8 mm and a constant temperature duration of 120 min (Figure 8a), K_0_ exhibits a non-monotonic response to increasing temperature. Relative to the ambient condition (193.90 kN/mm), the stiffness decreases sharply to 107.78 kN/mm at 100 °C, followed by a slight recovery at 200 °C (111.84 kN/mm), and then declines again at 300 °C. A similar temperature-dependent trend is observed for specimens with other wall thicknesses, although their stiffness evolution paths differ at the same temperature level, indicating a clear size-dependent response of K_0_ to thermal exposure. It should be noted that K_0_ is extracted from the initial portion of the load–displacement curve and may be influenced by seating/end-contact adjustment, closure of thermal microcracks, and the compliance of the test setup. Therefore, the observed non-monotonic variations in K_0_ with temperature likely reflect the combined effects of thermal degradation and measurement/setup-related factors.

Conditional on exposure temperature, the influence of wall thickness on initial stiffness is non-uniform (Figure 8b). At 100 °C, increasing the wall thickness from 5 mm to 10 mm leads to an approximately 43% increase in K_0_, suggesting that the confinement provided by thicker GFRP tubes remains effective at relatively low temperatures. However, at 150 °C and above, this enhancement becomes unstable. At 200 °C, for example, the specimen with a 5 mm wall thickness exhibits a higher initial stiffness than the 8 mm specimen, reflecting a deterioration of the stiffness contribution associated with increased wall thickness under elevated temperatures.

The effect of constant temperature duration on initial stiffness is also temperature-dependent (Figure 8c). For specimens with an 8 mm wall thickness, extending the exposure duration from 60 min to 120 min at 100 °C and 200 °C results in a noticeable reduction in K_0_, whereas at 150 °C the influence of duration is limited and no systematic degradation trend is observed. This behavior indicates that stiffness degradation is largely established during the early stage of thermal exposure.

Taken together, initial stiffness degradation of CFGFT stub columns is dominated by exposure temperature, while wall thickness and exposure duration primarily influence the rate and path of degradation. The stiffness benefit associated with increased wall thickness is confined to low and moderate temperature ranges and progressively diminishes at higher temperatures, whereas the role of exposure duration remains secondary and strongly temperature-dependent. These observations define the applicable boundary conditions for stiffness evaluation of CFGFT members after elevated-temperature exposure.

### 3.3. Load–Strain Response

To characterize the confinement behavior of circular GFRP tubes under axial compression, nine representative CFGFT short column specimens were selected for strain measurements. Axial and circumferential strains were simultaneously recorded at the mid-height section of the GFRP tube. It should be noted that the strain gauges were bonded to the outer tube surface after the heating–cooling process; although the post-heating surface condition may affect local bonding quality, the strain results are used here to indicate overall confinement-response trends.

**Figure 9:**
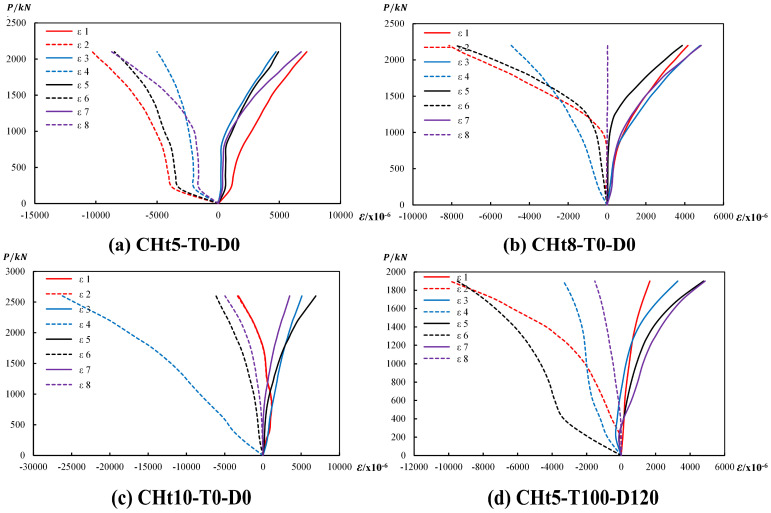

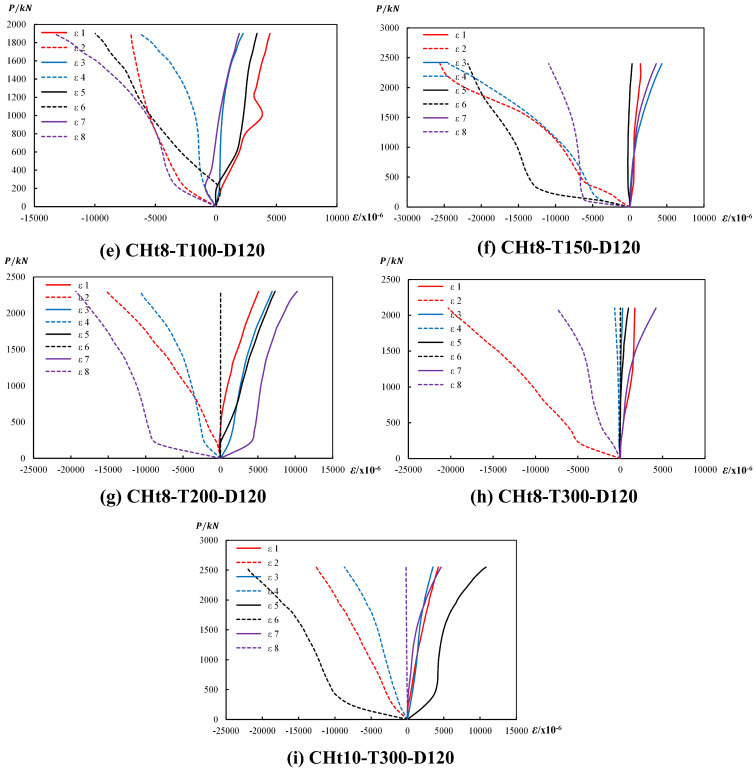
Axial load–circumferential strain curves of representative CFGFT short columns.

The axial load–strain curves are presented in Figure 9 and are used to evaluate the effects of GFRP tube wall thickness and exposure temperature on the confinement response of the specimens.

At ambient temperature, the axial load–circumferential strain response is clearly affected by the wall thickness of the GFRP tubes (Figure 9a–c). With an increase in wall thickness, the initial slope of the curves becomes steeper, indicating an enhancement in the axial stiffness of the members. Meanwhile, the circumferential strain corresponding to the ultimate load decreases as the wall thickness increases. This response suggests that thicker GFRP tubes provide stronger circumferential restraint to the core concrete, allowing the specimens to sustain higher axial loads while effectively suppressing transverse deformation.

With increasing temperature, the restraining behavior of the GFRP tubes exhibits a consistent degradation trend. Taking the specimens with a wall thickness of 8 mm as an example (Figure 9b,d–h), the initial tangent stiffness of the curves gradually decreases as temperature rises, reflecting the deterioration of the GFRP tube stiffness. In addition, the transition point from linear to nonlinear behavior shifts toward lower load levels, accompanied by a reduction in both axial load and circumferential strain at this stage. The ultimate load capacity and the corresponding peak circumferential strain further decrease with increasing temperature. At 300 °C, the specimens exhibit pronounced softening at relatively low load levels, and the development of circumferential strain is notably limited, indicating that the confinement effect provided by the GFRP tubes is substantially weakened under severe thermal exposure.

Under elevated-temperature exposure, the beneficial effect of increasing wall thickness can still be observed, although it becomes secondary to the overall thermal degradation. At the same temperature level, specimens with thicker GFRP tubes generally exhibit higher load–circumferential strain curves than their thin-walled counterparts (Figure 9d,i), indicating a higher residual confinement capacity after heating.

By contrast, the initial slopes of all high-temperature curves are markedly lower than those obtained at room temperature, regardless of wall thickness. This response reflects a generalized degradation of GFRP material stiffness induced by temperature, which dominates the early-stage mechanical behavior. Under these conditions, increased wall thickness mainly mitigates performance loss rather than providing an absolute enhancement.

From a strain-mechanism perspective, wall thickness improves axial performance by increasing the circumferential confinement stiffness of the GFRP tube, whereas elevated temperature progressively weakens this confinement by reducing material stiffness and triggering earlier loss of restraint. This interaction defines the boundary conditions under which confinement efficiency deteriorates and explains the reductions in load capacity and stiffness discussed in Section 3.2.

## 4. Analysis and Discussion

### 4.1. Influence Mechanism of Key Parameters on Mechanical Properties

The results presented in Section 3 demonstrate that the axial load-bearing capacity, initial stiffness, ductility, and failure mode of circular CFGFT short columns are all degraded to varying extents after exposure to elevated temperatures. This degradation does not originate from a single controlling factor, but rather from the combined effects of exposure temperature, GFRP tube wall thickness, and constant temperature duration.

Instead of reiterating the experimental observations, this section interprets the results from a mechanistic perspective. Emphasis is placed on temperature-induced deterioration of the GFRP tube material and the associated evolution of the confinement mechanism. By linking these material-level changes to the macroscopic responses observed in Section 3, the governing influence mechanisms of the key parameters are clarified.

Mechanistically, the temperature-driven reductions in residual strength and stiffness are primarily associated with thermo-mechanical weakening of the GFRP tube (e.g., matrix softening as the resin approaches/exceeds its glass transition region, reduced shear transfer and hoop stiffness) and thermally induced damage in the concrete core (e.g., moisture migration, microcracking, and stiffness loss). As the confinement efficiency decreases, the contribution of the GFRP tube to enhancing axial resistance and stiffness diminishes, leading to lower Nu and K_0_. Increasing tube wall thickness enhances confinement stiffness and hoop resistance and is therefore more effective at low-to-moderate temperatures; however, under severe thermal exposure, the incremental benefit attenuates because confinement performance becomes governed by the degraded material state. Within the investigated duration window (60–120 min) under the adopted heating–holding–cooling protocol, peak temperature remains the dominant driver of residual behavior, while duration plays a secondary role that can be masked by thermal non-uniformity and specimen-to-specimen variability.

#### 4.1.1. Dominant Role of GFRP Tube Material Degradation at Elevated Temperatures

The degradation of axial compressive performance in CFGFT short columns at elevated temperatures primarily stems from the temperature-sensitive deterioration of GFRP tube material properties. In GFRP composites, the glass transition temperature of the resin matrix typically lies within 100–200 °C. As the exposure temperature approaches or exceeds this range, the resin transitions from a glassy to a rubbery state, accompanied by marked reductions in elastic modulus and strength.

At the structural level, this material degradation manifests as a systematic decrease in ultimate load capacity and initial stiffness with increasing temperature (Section 3.2). Simultaneously, the elastic portion of the load–displacement response shortens, while post-peak softening becomes more pronounced. From the load–circumferential strain perspective (Section 3.3), the initial tangent stiffness is reduced, the nonlinear inflection point occurs at lower load levels, and the peak circumferential strain decreases. These features collectively indicate a progressive weakening of the effective circumferential confinement provided by the GFRP tube as temperature rises.

In addition to resin degradation, elevated temperatures impair the fiber–matrix interface, thereby weakening the synergistic action within the GFRP tube. Under reduced interfacial bonding, the tube becomes more susceptible to crack initiation and propagation along the fiber direction, which is consistent with the increased occurrence of longitudinal splitting failures (Mode 3) observed after high-temperature exposure (Section 3.1.2). The transition in surface appearance from discoloration to localized charring (Section 3.1.1) further reflects the onset and non-uniform nature of thermal degradation in the resin matrix.

#### 4.1.2. Evolution of Confinement Mechanism Under Wall Thickness–Temperature Coupling

The influence of GFRP tube wall thickness on the axial compressive behavior of CFGFT short columns is strongly temperature-dependent. Fundamentally, the contribution of increased wall thickness arises from enhanced circumferential confinement stiffness and strength, conditional on effective utilization of the GFRP material properties.

At ambient and low-to-moderate temperatures (approximately 100–150 °C), thicker GFRP tubes provide stronger lateral restraint to the core concrete, resulting in higher ultimate load capacity and initial stiffness (Section 3.2). Correspondingly, the load–circumferential strain responses show that the peak circumferential strain at ultimate load decreases with increasing wall thickness (Section 3.3), indicating more effective suppression of concrete lateral dilation.

When the temperature increases to 200–300 °C, degradation of GFRP stiffness and strength becomes pronounced, and the confinement mechanism deteriorates at earlier loading stages. Consequently, performance differences between specimens with different wall thicknesses are substantially reduced (Section 3.2). Although thicker-walled specimens retain a relative advantage, this advantage exists within an overall degraded performance regime and no longer translates into the absolute strengthening observed at lower temperatures. Consistently, the initial slopes of the load–circumferential strain curves for all high-temperature specimens are markedly lower than those at ambient temperature, indicating a generalized reduction in confinement stiffness governed by thermal effects.

The coupled effects of wall thickness and temperature are also reflected in failure patterns. Thin-walled specimens tend to exhibit global circumferential fracture at mid-height, whereas thick-walled specimens more frequently show localized end crushing. With increasing temperature, the proportion of longitudinal splitting failures increases (Section 3.1.2). These observations suggest that wall thickness governs the spatial distribution of confinement, while temperature-induced material and interfacial degradation amplifies the likelihood of unfavorable failure modes.

#### 4.1.3. Interpretation of Non-Monotonic Responses and Role of Constant Temperature Duration

Within the temperature range of 150–200 °C, the ultimate load capacity of several specimens does not decrease strictly monotonically, but instead exhibits limited fluctuations (Table 4). These variations are not consistently associated with specific wall thicknesses or exposure durations, nor do they form a repeatable trend. Quantitatively, comparing the reduction factor λ between 150 °C and 200 °C at fixed thickness and duration shows that five of six thickness–duration pairs vary only by approximately 2–12% (with inconsistent directions across the matrix). One pair (Ht = 10 mm, D = 60 min) exhibits a larger deviation (λ: 1.077 → 0.573; −46.8%), which coincides with a change in failure mode (Mode 1 → Mode 3), and is therefore treated as an isolated outlier within the single-specimen program.

Rather than indicating a stable strengthening mechanism, the limited non-monotonicity is more plausibly attributed to competing temperature-dependent processes and thermal non-uniformity. In this intermediate temperature range, concrete conditioning associated with moisture migration/drying may partially offset thermally induced microcracking, while the confinement efficiency of the GFRP tube may degrade in a nonlinear manner as the polymer matrix approaches its softening region, leading to small fluctuations in residual capacity. Moreover, because internal temperatures through the tube thickness and along the specimen height were not monitored, different thermal gradients/time lags may lead to different effective internal temperatures even under the same furnace set-point, which can amplify specimen-to-specimen differences.

Based on the overall experimental evidence, such behavior is more reasonably attributed to inherent variability in material degradation and confinement evolution under thermal exposure, rather than to any systematic enhancement or transition in load-resisting mechanisms. Under the test conditions considered in this study, there is insufficient evidence to interpret these fluctuations as a stable or reproducible “transient stabilization” phenomenon.

Regarding constant temperature duration, comparisons between specimens exposed for 60 min and 120 min at the same temperature show no consistent differences in ultimate load capacity, initial stiffness, or load-displacement behavior (Section 3.2). The absence of consistent differences in these parameters suggests that the exposure duration, within the investigated time scale, does not significantly affect the structural performance of CFGFT short columns. Within the investigated time scale, peak temperature therefore dominates the post-fire residual performance, while exposure duration plays a secondary role. Accordingly, the effect of exposure duration on ultimate load capacity, initial stiffness, and ductility is generally limited within the present program. It should be noted that some individual 60–120 min specimen pairs exhibit relatively large differences; however, the direction and magnitude of these differences are not consistent across temperatures and wall thicknesses and therefore do not constitute a repeatable duration-dependent trend. Such local deviations are likely amplified by specimen-to-specimen variability and thermal non-uniformity (i.e., different effective internal temperatures/time lags under the same furnace set-point).

#### 4.1.4. Reassessment of Load Capacity–Ductility Relationship and Engineering Implications

The experimental results indicate that the ductility coefficient μ does not degrade in parallel with the ultimate load-carrying capacity after elevated temperature exposure (Section 3.2.2). In several cases, particularly for thin-walled specimens, relatively high μ values are observed despite substantial reductions in ultimate strength. This decoupling suggests that ductility retention alone is not a reliable indicator of post-fire structural safety. This outcome is partly related to the adopted definition μ = Δ_u_/Δ_y_, because both Δ_y_ and Δ_u_ may decrease after heating and their ratio can remain within a narrow range, while the absolute resistance is significantly reduced.

For thin-walled members, degradation of the GFRP tube at elevated temperature leads to an earlier loss of effective confinement, causing the member to enter a nonlinear deformation stage dominated by the core concrete at lower load levels. Under these conditions, deformation continues to accumulate, resulting in moderate μ values, while the ultimate load capacity—and thus the upper bound of safe load resistance—is significantly reduced. The apparent preservation of ductility therefore reflects a change in deformation mechanism rather than sustained load-bearing performance.

From an engineering standpoint, post-fire assessment of CFGFT members should not rely solely on ductility indices. Residual load-bearing capacity and deformation capacity must be evaluated jointly, particularly for thin-walled members exposed to high temperatures, to avoid unconservative assessments of structural safety. In practice, residual capacity (e.g., λ = N_u_/N_0_) should be treated as the primary safety-related indicator, while K_0_ and μ provide complementary information on stiffness degradation and deformation development (warning capacity). Therefore, μ should be used as a supplementary descriptor rather than a stand-alone acceptance criterion.

### 4.2. Prediction Model for Ultimate Axial Load Capacity

Based on the experimental results presented in Section 3, the residual axial compressive capacity of circular CFGFT short columns after elevated temperature exposure is governed primarily by the exposure temperature T and the GFRP tube wall thickness Ht Within the investigated time range (60–120 min), the influence of constant temperature duration is comparatively limited and does not show a systematic contribution to strength degradation.

To facilitate rapid post-fire assessment of axial load-carrying capacity, a load reduction factor Kr is introduced to quantify the degradation induced by elevated temperature. The reduction factor is defined as the ratio between the experimentally measured ultimate axial capacity after thermal exposure, Nu, and the corresponding theoretical ultimate axial capacity at ambient temperature, *N*_0_:

The ambient-temperature axial capacity *N*_0_ is evaluated using confinement-based formulations originally developed and validated for CFST stub columns under axial compression (e.g., Yang and Han [27,28]). Although these expressions were calibrated for steel tubes, they are adopted here as a conservative for defining *K*_r_ = *N_u_*/*N*_0_, noting that GFRP confinement differs from steel confinement (elastic–rupture response without yielding).

*K*_r_ = *N*_u_/*N*_0_ (2)

*N*_0_ = *A*_sc_ × *f*_sc_ (3)

*f*_sc_ = (1.14 + 1.02*ξ*) × *f*_c_ (4)

*A*_sc_ = *A*_s_ + *A*_c_ (5)

*ξ* = (*A*_s_ × *f*_s_)/(*A*_c_ × *f*_c_) (6)

Based on the test data of 27 specimens (Table 5), the experimental load reduction factor *K_r_*_,exp_ was calculated for each specimen. For the three ambient control specimens, *K_r_*_,exp_ ranges from 1.019 to 1.298 (i.e., slightly greater than 1.0), indicating a conservative tendency of Equations (3)–(6) when used as an ambient reference baseline in the present test matrix.

For specimens exposed to elevated temperatures, *K_r_*_,exp_ is distributed over a wider range of 0.588–1.288, exhibiting noticeably greater dispersion. It should be noted that cases with *K_r_*_,exp_ > 1.0 do not indicate a systematic post-fire strength gain; rather, they reflect the combined effects of experimental scatter and the conservative nature/limited transferability of the analytical baseline *N*_0_ to GFRP-confined concrete. This increased scatter reflects the combined effects of varying degrees of material degradation and differences in failure modes on the residual load-bearing capacity of the GFRP tube after thermal exposure. Such behavior is consistent with the non-monotonic strength variation and failure mode transitions observed in Section 3.

Considering the nonlinear nature of temperature-induced degradation and the gain effect associated with GFRP tube wall thickness, a normalized binary quadratic regression model is proposed to predict the reduction factor *K_r_*:

*K_r_*(*T*,*H_t_*) = 1 + *a*_1_ · (*T*/300) + *a*_2_ · (*H_t_*/10) + *a*_3_ · (*T*/300)^2^ + *a*_4_ · (*T*/300)(*H_t_*/10) (7)

where 300 °C and 10 mm represent the maximum temperature and wall thickness in the present test program and are used as normalization constants to improve numerical stability and regression robustness.

Using the data from 24 specimens exposed to elevated temperatures, the regression coefficients were determined by least-squares fitting. The resulting regression coefficients and statistical indicators are summarized in Table 6. All regression terms exhibit *p*-values lower than 0.001, indicating statistically significant contributions. The fitted model achieves a coefficient of determination *R*^2^ = 0.901 (adjusted *R*^2^ = 0.887), with RMSE = 0.048 in *K_r_* and MAE = 7.2%, demonstrating a reasonable trend-level predictive capability for rapid engineering assessment.

Substituting the fitted coefficients into Equation (7), the final prediction model is expressed as follows:

*K_r_*(*T*,*H_t_*) = 1 − 0.425 · (*T*/300) + 0.183 · (*H_t_*/10) − 0.328 · (*T*/300)^2^ + 0.095 · (*T*/300)(*H_t_*/10) (8)

**Figure 10:**
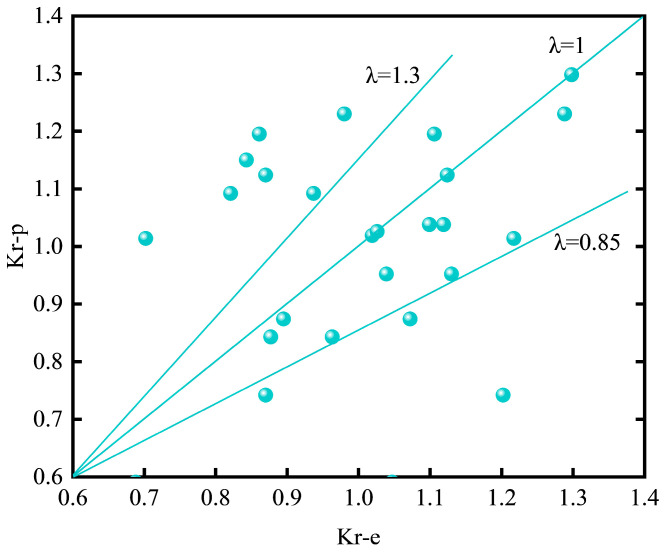
Comparison between predicted and experimental reduction factors, where dots represent test results and solid lines denote the y = x reference line and model bounds.

The comparison between predicted and experimental reduction factors is shown in Figure 10. Most data points cluster closely around the y = x reference line, indicating that the proposed model captures the overall degradation trend with respect to temperature and wall thickness. Larger deviations are observed for a limited number of specimens (e.g., Ht = 10 mm at T = 200 °C), which can be attributed to experimental dispersion associated with heterogeneous material degradation and variations in failure mode, as well as to the simplified form of the regression model in which secondary influences (e.g., exposure duration and effective internal thermal gradients) are not explicitly included. Accordingly, the presence of some *K_r_*_,exp_ > 1.0 data points should be interpreted as baseline conservatism and experimental dispersion rather than true “post-fire strengthening”, and it does not alter the overall degradation trend captured by the model.

From a mechanistic perspective, Equation (8) reflects the dominant thermal degradation of confinement efficiency with increasing temperature, the strengthening contribution of increased wall thickness at lower temperatures, and the progressive attenuation of this benefit under severe thermal damage conditions.

The proposed reduction factor model is intended for engineering assessment of the residual axial compressive capacity of circular CFGFT short columns after fire, provided that the peak exposure temperature and GFRP tube wall thickness are known. Within the investigated parameter ranges (ambient–300 °C, Ht = 5–10 mm, duration 60–120 min), the model offers a reasonable balance between predictive accuracy and practical simplicity. When individual predictions are used for decision-making, it is recommended to interpret the estimated *K_r_* together with observed failure characteristics and to adopt conservative judgment for cases showing pronounced scatter. It should be noted that the model was calibrated using the present dataset and has not yet been independently validated against external datasets; therefore, its use outside the investigated ranges or under different fire curves/material systems should be treated with caution. For applications requiring higher precision, additional parameters such as exposure duration, effective internal thermal gradients or damage-mode-dependent formulations may be incorporated, or segmented models based on temperature ranges may be adopted.

## 5. Conclusions

Based on axial compression tests on 27 circular GFRP tube-confined concrete (CFGFT) short columns after elevated temperature exposure, the effects of temperature, GFRP tube wall thickness, and constant temperature duration on post-fire mechanical performance were investigated. The confinement mechanism after heating was further examined through strain responses and failure characteristics. The main conclusions are as follows:

1. Temperature governs post-fire degradation.

Elevated temperature is the primary factor controlling the degradation of axial compressive performance. Both the ultimate axial capacity and initial stiffness decrease with increasing temperature, with a pronounced deterioration observed in the range of 200–300 °C. Within the investigated duration range of 60–120 min, the effect of constant temperature exposure time is secondary and does not exhibit a stable or systematic influence on residual capacity or stiffness, indicating that peak temperature dominates post-fire performance at this time scale.

2. The strengthening role of wall thickness is temperature-dependent.

Increasing the GFRP tube wall thickness enhances axial capacity and initial stiffness under ambient and low-to-moderate temperature conditions. As the exposure temperature exceeds approximately 200 °C, degradation of GFRP material properties leads to an earlier loss of effective confinement, and the performance differences between specimens with different wall thicknesses diminish. Under severe thermal exposure, wall thickness provides only a relative advantage rather than preserving absolute load-carrying capacity.

3. Failure modes reflect coupled effects of temperature and wall thickness.

Thin-walled specimens mainly fail through global circumferential fracture at mid-height, whereas thick-walled specimens tend to exhibit localized end crushing. With increasing temperature, longitudinal splitting becomes increasingly prevalent, indicating that thermal degradation of the GFRP material and fiber–matrix interface weakens confinement continuity and structural integrity.

4. Ductility retention does not imply strength preservation.

The ductility coefficient does not decrease synchronously with residual axial capacity after elevated temperature exposure. In particular, thin-walled specimens may retain relatively high calculated ductility values despite a substantial reduction in ultimate load capacity. This decoupling indicates that ductility alone is insufficient for post-fire safety evaluation, and residual strength and deformation capacity should be assessed jointly to avoid unconservative judgments.

5. A practical residual capacity model is established.

A residual axial-capacity model is proposed using a reduction factor *K_r_* incorporating peak temperature and GFRP tube wall thickness. The model shows good agreement with the experimental results (R_2_ = 0.901) and provides a practical tool for rapid post-fire assessment within the investigated range. Future refinement may incorporate secondary variables such as exposure duration and failure-mode effects. Future work is also recommended to instrument specimens with thermocouples to quantify internal temperature histories and thermal gradients and to evaluate CFGFT members under other representative fire heating curves (e.g., parametric fire curves and hydrocarbon fire curves) to broaden the applicability of the present findings.

## Figures and Tables

**Table 1:** Design Parameters of Concrete-Filled GFRP Tube (CFGFT) Short Column Specimens.

Specimen ID	H_t_ (mm)	D_0_ (mm)	H (mm)	H/D_0_	T (°C)	D (min)
C-Ht5-T0-D0	5	200	450	2.25	20	0
C-Ht8-T0-D0	8	200	450	2.25	20	0
C-Ht10-T0-D0	10	200	450	2.25	20	0
C-Ht5-T100-D60	5	200	450	2.25	100	60
C-Ht5-T100-D120	5	200	450	2.25	100	120
C-Ht5-T150-D60	5	200	450	2.25	150	60
C-Ht5-T150-D120	5	200	450	2.25	150	120
C-Ht5-T200-D60	5	200	450	2.25	200	60
C-Ht5-T200-D120	5	200	450	2.25	200	120
C-Ht5-T300-D60	5	200	450	2.25	300	60
C-Ht5-T300-D120	5	200	450	2.25	300	120
C-Ht8-T100-D60	8	200	450	2.25	100	60
C-Ht8-T100-D120	8	200	450	2.25	100	120
C-Ht8-T150-D60	8	200	450	2.25	150	60
C-Ht8-T150-D120	8	200	450	2.25	150	120
C-Ht8-T200-D60	8	200	450	2.25	200	60
C-Ht8-T200-D120	8	200	450	2.25	200	120
C-Ht8-T300-D60	8	200	450	2.25	300	60
C-Ht8-T300-D120	8	200	450	2.25	300	120
C-Ht10-T100-D60	10	200	450	2.25	100	60
C-Ht10-T100-D120	10	200	450	2.25	100	120
C-Ht10-T150-D60	10	200	450	2.25	150	60
C-Ht10-T150-D120	10	200	450	2.25	150	120
C-Ht10-T200-D60	10	200	450	2.25	200	60
C-Ht10-T200-D120	10	200	450	2.25	200	120
C-Ht10-T300-D60	10	200	450	2.25	300	60
C-Ht10-T300-D120	10	200	450	2.25	300	120

**Table 2:** Mix Proportion of C30 Concrete and 28-Day Cubic Compressive Strength.

Parameter	Cement (kg/m^3^)	Sand (kg/m^3^)	Coarse Aggregate (kg/m^3^)	Water (kg/m^3^)	Specimen Name	f_cu_ (MPa)	
					C30-1	32.4	
C30	450	600	1192	183	C30-2	33.5	33.2
					C30-3	33.7	

**Table 3:** GFRP Material Properties.

Material Name	Relative Density	Longitudinal Compressive Modulus (GPa)	Coefficient of Thermal Expansion (10^6^/°C)	Elongation (%)	Longitudinal Compressive Strength (MPa)	Transverse Tensile Strength (MPa)
GFRP	2.55	17	5	4.8	192	207

**Table 4:** Summary of key axial compression performance parameters for circular CFGFT stub columns.

Specimen ID	N_u_ (kN)	N_y_ (kN)	λ	K_0_ (kN/mm)	Δ_u_ (mm)	Δ_y_ (mm)	μ	Failure Mode
C-Ht5-T0-D0	2128.00	1702.40	1.000	136.10	15.64	13.15	1.19	Mode 2
C-Ht8-T0-D0	2254.00	1803.68	1.000	193.90	11.63	9.19	1.27	Mode 1
C-Ht10-T0-D0	2698.00	2158.40	1.000	117.25	23.01	19.68	1.17	Mode 1
C-Ht5-T100-D60	1151.00	920.96	0.541	82.28	13.99	12.24	1.14	Mode 3
C-Ht5-T100-D120	1994.00	1595.20	0.937	100.73	19.80	17.36	1.14	Mode 3
C-Ht5-T150-D60	1468.00	1174.40	0.690	104.69	14.02	12.63	1.11	Mode 3
C-Ht5-T150-D120	1757.00	1405.92	0.826	109.83	16.00	14.04	1.14	Mode 1
C-Ht5-T200-D60	1426.00	1141.44	0.670	91.26	15.64	13.73	1.14	Mode 1
C-Ht5-T200-D120	1970.00	1576.48	0.926	128.45	15.34	12.73	1.20	Mode 3
C-Ht5-T300-D60	1717.00	1373.92	0.807	128.72	13.34	11.41	1.17	Mode 2
C-Ht5-T300-D120	1127.00	902.24	0.530	143.98	7.83	6.75	1.16	Mode 3
C-Ht8-T100-D60	2487.00	1990.24	1.103	146.91	16.93	13.69	1.24	Mode 1
C-Ht8-T100-D120	1925.00	1540.64	0.854	107.78	17.87	15.26	1.17	Mode 3
C-Ht8-T150-D60	2432.00	1946.08	1.079	121.95	19.95	16.70	1.20	Mode 2
C-Ht8-T150-D120	2477.00	1982.24	1.099	132.80	18.66	15.35	1.22	Mode 2
C-Ht8-T200-D60	2501.00	2000.80	1.110	191.23	13.08	10.15	1.29	Mode 3
C-Ht8-T200-D120	2300.00	1840.16	1.020	111.84	20.57	17.96	1.14	Mode 1
C-Ht8-T300-D60	1940.00	1552.48	0.861	174.51	11.12	9.28	1.20	Mode 1
C-Ht8-T300-D120	2131.00	1705.44	0.945	117.25	18.18	15.43	1.18	Mode 2
C-Ht10-T100-D60	3385.00	2708.00	1.255	148.93	22.73	18.02	1.26	Mode 2
C-Ht10-T100-D120	2575.00	2060.16	0.954	143.88	17.90	14.83	1.21	Mode 2
C-Ht10-T150-D60	2906.00	2325.44	1.077	140.30	20.72	17.05	1.21	Mode 1
C-Ht10-T150-D120	2263.00	1810.40	0.839	111.13	20.36	17.31	1.18	Mode 3
C-Ht10-T200-D60	1546.00	1237.28	0.573	105.05	14.72	12.26	1.20	Mode 3
C-Ht10-T200-D120	2217.00	1773.60	0.822	187.24	11.84	9.54	1.24	Mode 1
C-Ht10-T300-D60	2462.00	1970.24	0.912	167.87	14.67	12.17	1.20	Mode 1
C-Ht10-T300-D120	2158.00	1727.04	0.800	189.30	11.40	8.93	1.27	Mode 2

Note: λ = N_u_/N_0_, where N_0_ and Nu denote the ultimate axial load capacities of the circular CFGFT short column at ambient temperature and after elevated temperature exposure, respectively. Some specimens exhibit λ > 1, which can be attributed to localized variations in thermal exposure and material heterogeneity. These specimens showed higher than expected post-heating capacity at peak load, but such fluctuations are expected in high-temperature fire tests and do not affect the overall trends observed. The deviations are inherent to the experimental setup and the nature of fire exposure.

**Table 5:** Experimental axial compressive capacity and reduction factors.

Specimen ID	N_u,exp_ (kN)	N_0_ (kN)	K_r,exp_	K_r,pred_	N_u,pred_ (kN)	Error (%)
C-Ht5-T0-D0	2128.0	1638.8	1.298	1.298	2128.0	0.0
C-Ht8-T0-D0	2254.0	2212.5	1.019	1.019	2254.0	0.0
C-Ht10-T0-D0	2698.0	2628.5	1.026	1.026	2698.0	0.0
C-Ht5-T100-D60	1151.0	1638.8	0.702	1.014	1661.2	44.3
C-Ht5-T100-D120	1994.0	1638.8	1.217	1.014	1661.2	−16.7
C-Ht5-T150-D60	1468.0	1638.8	0.895	0.874	1432.1	−2.4
C-Ht5-T150-D120	1757.0	1638.8	1.072	0.874	1432.1	−18.5
C-Ht5-T200-D60	1426.0	1638.8	0.870	0.742	1215.3	−14.8
C-Ht5-T200-D120	1970.0	1638.8	1.202	0.742	1215.3	−38.3
C-Ht5-T300-D60	1717.0	1638.8	1.047	0.591	968.5	−43.6
C-Ht5-T300-D120	1127.0	1638.8	0.688	0.591	968.5	−14.1
C-Ht8-T100-D60	2487.0	2212.5	1.124	1.124	2487.0	0.0
C-Ht8-T100-D120	1925.0	2212.5	0.870	1.124	2487.0	29.2
C-Ht8-T150-D60	2432.0	2212.5	1.099	1.038	2296.8	−5.6
C-Ht8-T150-D120	2477.0	2212.5	1.119	1.038	2296.8	−7.3
C-Ht8-T200-D60	2501.0	2212.5	1.130	0.952	2106.0	−15.8
C-Ht8-T200-D120	2300.0	2212.5	1.039	0.952	2106.0	−8.4
C-Ht8-T300-D60	1940.0	2212.5	0.877	0.843	1865.0	−3.9
C-Ht8-T300-D120	2131.0	2212.5	0.963	0.843	1865.0	−12.5
C-Ht10-T100-D60	3385.0	2628.5	1.288	1.230	3234.5	−4.4
C-Ht10-T100-D120	2575.0	2628.5	0.980	1.230	3234.5	25.6
C-Ht10-T150-D60	2906.0	2628.5	1.106	1.195	3140.6	8.1
C-Ht10-T150-D120	2263.0	2628.5	0.861	1.195	3140.6	38.8
C-Ht10-T200-D60	1546.0	2628.5	0.588	1.150	3022.8	95.5
C-Ht10-T200-D120	2217.0	2628.5	0.843	1.150	3022.8	36.3
C-Ht10-T300-D60	2462.0	2628.5	0.937	1.092	2871.1	16.6
C-Ht10-T300-D120	2158.0	2628.5	0.821	1.092	2871.1	33.0

Note: N*_u,p_* = K*_r,p_* × N0; Error = (N*_u,p_* − N*_u,e_*)/N*_u,e_* × 100%.

**Table 6:** Coefficients and accuracy of the regression model.

Parameter	Value	Std. Error	*p*-Value
a_1_ (T/300)	−0.425	0.035	<0.001
a_2_ (Ht/10)	0.183	0.028	<0.001
a_3_ (T/300)^2^	−0.328	0.041	<0.001
a_4_ (T/300) × (Ht/10)	0.095	0.019	<0.001
R^2^	0.901	—	—
MAE (%)	7.2	—	—

## Data Availability

The original contributions presented in this study are included in the article. Further inquiries can be directed to the corresponding authors.
